# Identification of Novel Biomarkers for Drug Hypersensitivity After Sequencing of the Promoter Area in 16 Genes of the Vitamin D Pathway and the High-Affinity IgE Receptor

**DOI:** 10.3389/fgene.2019.00582

**Published:** 2019-06-25

**Authors:** Gemma Amo, Manuel Martí, Jesús M. García-Menaya, Concepción Cordobés, José A. Cornejo-García, Natalia Blanca-López, Gabriela Canto, Inmaculada Doña, Miguel Blanca, María José Torres, José A. G. Agúndez, Elena García-Martín

**Affiliations:** ^1^University Institute of Molecular Pathology Biomarkers, UEx, Cáceres, Spain; ^2^ARADyAL Instituto de Salud Carlos III, Cáceres, Spain; ^3^Allergy Service, Badajoz University Hospital, Badajoz, Spain; ^4^ARADyAL Instituto de Salud Carlos III, Badajoz, Spain; ^5^Allergy Service, Mérida Hospital, Badajoz, Spain; ^6^ARADyAL Instituto de Salud Carlos III, Cáceres, Spain; ^7^Research Laboratory, IBIMA, Regional University Hospital of Málaga, UMA, Málaga, Spain; ^8^ARADyAL Instituto de Salud Carlos III, Cáceres, Spain; ^9^Allergy Service, Infanta Leonor University Hospital, Madrid, Spain; ^10^ARADyAL Instituto de Salud Carlos III, Madrid, Spain; ^11^Allergy Unit, IBIMA, Regional University Hospital of Málaga, UMA, Málaga, Spain; ^12^ARADyAL Instituto de Salud Carlos III, Málaga, Spain

**Keywords:** Next-Generation Sequencing (NGS), vitamin D, high-affinity IgE receptor (FCεRI), NSAIDs (non-steroidal anti-inflammatory drugs), beta-lactam antibiotic, drugs hypersensitivity reactions, allergic rhinitis, asthma

## Abstract

The prevalence of allergic diseases and drug hypersensitivity reactions (DHRs) during recent years is increasing. Both, allergic diseases and DHRs seem to be related to an interplay between environmental factors and genetic susceptibility. In recent years, a large effort in the elucidation of the genetic mechanisms involved in these disorders has been made, mostly based on case-control studies, and typically focusing on isolated SNPs. These studies provide a limited amount of information, which now can be greatly expanded by the complete coverage that Next Generation Sequencing techniques offer. In this study, we analyzed the promoters of sixteen genes related to the Vitamin D pathway and the high-affinity IgE receptor, including *FCER1A, MS4A2, FCER1G, VDR, GC, CYP2R1, CYP27A1, CYP27B1, CYP24A1, RXRA, RXRB, RXRG, IL4, IL4R, IL13*, and *IL13RA1*. The study group was composed of patients with allergic rhinitis plus asthma (AR+A), patients with hypersensitivity to beta-lactams (BLs), to NSAIDs including selective hypersensitivity (SH) and cross-reactivity (CR), and healthy controls without antecedents of atopy or adverse drug reactions. We identified 148 gene variations, 43 of which were novel. Multinomial analyses revealed that three SNPs corresponding to the genes *FCER1G* (rs36233990 and rs2070901), and *GC* (rs3733359), displayed significant associations and, therefore, were selected for a combined dataset study in a cohort of 2,476 individuals. The strongest association was found with the promoter *FCER1G* rs36233990 SNP that alters a transcription factor binding site. This SNP was over-represented among AR+A patients and among patients with IgE-mediated diseases, as compared with control individuals or with the rest of patients in this study. Classification models based on the above-mentioned SNPs were able to predict correct clinical group allocations in patients with DHRs, and patients with IgE-mediated DHRs. Our findings reveal gene promoter SNPs that are significant predictors of drug hypersensitivity, thus reinforcing the hypothesis of a genetic predisposition for these diseases.

## Introduction

The prevalence of atopy, allergic diseases, and drug hypersensitivity reactions (DHRs) is increasing worldwide. In Europe, studies have estimated a prevalence of 20–25% allergic diseases in adults, with many young people being unaware of their disease (Linneberg, 2011; Kruse and Vanijcharoenkarn, 2018), which means an important economic impact for healthcare (European Commission, 2008; Bouvy et al., 2015) reaching an amount from €55 to €151 billion per year in European Union, including indirect costs related to the absence or reduced productivity at work (Zuberbier et al., 2014; Kruse and Vanijcharoenkarn, 2018). Due to their complexity, it is difficult to understand the specific mechanisms and molecules involved in the development of these diseases or to establish a way to prevent or reduce them. Allergic rhinitis (AR) reduces the quality of life by affecting sleep, school, work productivity, and social life. AR is an immunoglobulin E (IgE) mediated inflammatory disease, which is associated with other inflammatory diseases such as asthma. It has been estimated that around 20% of people in the USA and Europe suffer from allergic rhinitis (Durham et al., 2012; Ozdoganoglu and Songu, 2012; Rondon et al., 2017). Taking this into consideration, AR has been classified as a major chronic respiratory disease (Brozek et al., 2017). Drug hypersensitivity reactions (DHR) account for, ~3 to 6% of all hospital admissions. These reactions occur in 10 to 15% of hospitalized patients (Gomes and Demoly, 2005; Szczeklik and Nizankowska-Mogilnicka, 2009; Doña et al., 2014). Beta-lactam antibiotics (BLs) are the most common cause of DHRs mediated by specific immunological mechanisms (Antúnez et al., 2006; Doña et al., 2012, 2014) and, although the mechanisms of how the immune system recognizes these drugs are not fully determined, BLs are considered the classical model of this type of reactions (Blanca et al., 2009). Together with BLs, non-steroidal anti-inflammatory drugs (NSAIDs) are account for the vast majority of DHRs (Gomes et al., 2004; Messaad et al., 2004; Chen et al., 2012; Doña et al., 2012), but, in this case, these DHRs are not exclusively mediated by specific immunological mechanisms (selective hypersensitivity), involving a response to a single drug and good tolerance to other chemically unrelated NSAIDs (Canto et al., 2009; Cornejo-Garcia et al., 2009); but also by nonspecific immunological mechanisms (cross-reactions), which can be caused by more than one chemically unrelated NSAIDs (Agúndez et al., 2012; Kowalski et al., 2013).

Recent investigation proposes the vitamin D pathway among putative factors linked to allergic diseases, because of its important role in immune system (Veldman et al., 2000; Cantorna et al., 2015) and its direct relation with allergic diseases (Black and Scragg, 2005; Camargo et al., 2007; Benson et al., 2012; Suaini et al., 2015). There are many molecules involved in the vitamin D pathway: hydroxylases from CYP450 family, such as CYP27A1, CYP27B1, CYP2R1, and CYP24A1; the vitamin D binding protein (GC) that acts like a transporter, the vitamin D receptor (VDR), the retinoid receptor X (RXR) and interleukins which participate in downstream pathway (IL4 and IL13). In addition, there are other target molecules and signaling pathways, which could be involved in allergic mechanisms, such as the high-affinity IgE Receptor (FCεRI), which plays a key role in allergic reactions. This receptor is stimulated by IgE, triggering mast cells and basophils activation, and the consequent release of inflammatory mediators. In human mast cells and basophils, FCεRI consist of a heterotetramer composed by three subunits: FCεRIα, the ligand-binding subunit which is encoded by *FCER1A* gene; FCεRIβ, a signal-augmenting subunit encoded by *MS4A2*; and FCεRIγ, a signal-transducing subunit that is presented like a dimer and it is encoded by *FCER1G* (Kinet, 1999; Potaczek and Kabesch, 2012). Elevated levels of IgE have been detected in atopic conditions like allergic rhinitis, asthma, atopic dermatitis, anaphylaxia (Platts-Mills, 2001; Wallace et al., 2008) thus making FCεRI a plausible target molecule in the study of the mechanisms involved in the development and in the clinical presentation of allergy.

It could be hypothesized that variations related to expression and/or function in genes of the vitamin D signaling pathways or FCεRI might modify the risk of developing rhinitis or DHRs, and/or the presentation of clinical manifestations of these reactions. As a matter of fact, several studies demonstrated an association between different allergic diseases, including DHRs, and polymorphisms in these genes (Poon et al., 2004; Raby et al., 2004; Donfack et al., 2005; Bossé et al., 2009; Saadi et al., 2009; Pillai et al., 2011; Micheal et al., 2013; Berenguer et al., 2014; Amo et al., 2016a; Narozna et al., 2016). Several studies addressed the putative impact of exonic and intronic SNPs within the above-mentioned genes and the risk of allergic diseases and/or DHR (Wjst, 2005; Wjst et al., 2006; Battle et al., 2007; Arshad et al., 2008; Sadeghnejad et al., 2008; Weidinger et al., 2008; Black et al., 2009; Bossé et al., 2009; Ferreira et al., 2009; Knutsen et al., 2010; Li et al., 2010, 2012, 2014, 2016; Michel et al., 2010; Moffatt et al., 2010; Cooper et al., 2011; Joubert et al., 2011; Liu et al., 2011; Lu et al., 2011; Park et al., 2011; Paternoster et al., 2011; Pillai et al., 2011; Burkhardt et al., 2012; Choi et al., 2012; Granada et al., 2012; Lasky-Su et al., 2012; Ramasamy et al., 2012; Robinson et al., 2012; Zhou et al., 2012; Anderson et al., 2013; Hur et al., 2013; Ismail et al., 2013; Movahedi et al., 2013; Potaczek et al., 2013; Sharma et al., 2014; Yang et al., 2014; Kumar et al., 2015; Papadopoulou et al., 2015; Pino-Yanes et al., 2015; Tian et al., 2015; Amo et al., 2016a,b; Han et al., 2016; Karaca et al., 2016; Narozna et al., 2016; Overton et al., 2016; Ådjers et al., 2017; Ashley et al., 2017; Park and Tantisira, 2017; Sun et al., 2017; Xu et al., 2017; Zhang et al., 2017; Zhao et al., 2017). However, there is little information about SNPs located in the promoters of these genes, which might have functional consequences.

In an attempt to identifying genetic susceptibility factors associated with allergy and/or DHRs, that may provide novel information to gain a better understanding of these pathologies, we carried out an exhaustive analysis of genetic variations situated in the promoter region of the mentioned genes by using Next Generation Sequencing (NGS) in patients with allergic rhinitis plus asthma (AR+A), BLs hypersensitivity, selective NSAIDs hypersensitivity (SH) and cross-reactions to NSAIDs (CR), as well as in healthy control individuals. The genes included in the study were *FCER1A, MS4A2, FCER1G, VDR, GC, CYP2R1, CYP27A1, CYP27B1, CYP24A1, RXRA, RXRB, RXRG, IL4, IL4R, IL13*, and *IL13RA1*. In addition, we also analyzed the interaction of genetic and non-genetic factors, such as age, gender, and antecedents of atopy, in the risk of developing these diseases.

## Patients And Methods

### Study Population

A total cohort of 2,476 individuals participated in this study. All were Caucasian Spanish individuals. These included 406 healthy controls without antecedents of atopy or adverse drug reactions, 528 patients with AR+A, 561 individuals with BLs hypersensitivity, 668 patients with NSAIDs cross-reactivity (CR), and 313 selective hypersensitivity patients (SH) which were single-NSAIDs responders. Written consent for participation was obtained for all participants. Patients were recruited at Hospitals participating in the study. All the patients who were invited to participate in the study agreed to do so. Control individuals were selected among students and staff in the University and Hospitals participating in the study. Characteristics of the study groups are summarized in Table 1. The diagnosis was carried out as described elsewhere (García-Martín et al., 2007; Doña et al., 2011; Amo et al., 2016a; Lacombe-Barrios, 2018). The protocol for this study was in accordance with the Declaration of Helsinki and its subsequent revisions and was approved by the respective Ethics Committees of the participating Hospitals.

**Table 1:** Characteristics of the participants.

	**Healthy controls (*n* = 406)**	**Patients with AR+A (*n* = 528)**	**Patients with BLs hypersensitivity (*n* = 561)**	**Patients with NSAIDs cross-reactions (CR) (*n* = 668)**	**Patients with NSAIDs selective hypersensitivity (SH) (*n* = 313)**
Women. *n* (%)	253 (62.3%)	292 (55.3%)	318 (56.7%)	390 (58.4%)	204 (65.2%)
Age + sd (range)	22.1 ± 4.7 (20–58)	32.4 ± 14.2 (14–79)	46.7 ± 14.5 (4–91)	41.8 ± 15.3 (5–92)	45.5 ± 16.0 (5–82)
Antecedents of atopy	0	100%	23.3%	20.8%	23.3%

To get a further analysis of the sample, we put together some of the groups of patients which share a specific characteristic. Thus, we defined three new groups of study: “DHR group,” were we included all the patients with DHR: namely, patients with hypersensitivity to BLs and NSAIDs (both, CR and SH); “DHR-IgE Group,” which comprises selective hypersensitivity to BLs and SH; and ”IgE Mediated Group,” where we included all the IgE-mediated reactions (AR+A, BLs and SH).

#### Identification of Novel Variants Using NGS

A subset of participants were selected for this phase. A total cohort of 175 individuals participated in this NGS analysis. These included 22 healthy controls without antecedents of atopy or adverse drug reactions, 22 patients with AR+A, 43 individuals with BLs hypersensitivity, 41 patients with NSAIDs cross-reactivity (CR), and 46 selective hypersensitivity patients (SH) which were single-NSAIDs responders. Characteristics of the participants are summarized in Table S1. Genomic DNA was obtained from leukocytes and purified according to standard procedures. DNA samples were analyzed by NGS after specific enrichment based on the Haloplex design. Details of the areas sequenced are shown in Table S2. DNA was digested with restriction enzymes specific for this design (Haloplex, Agilent, Santa Clara, CA, USA), followed by hybridization with specific probes, DNA circularization and selection of the target areas, according to the protocol supplied. Sequencing was carried out in a MiSeq sequencer (Illumina, San Diego, CA, USA) using the pair end format. The coverage was always higher than that recommended by the manufacturer (23.7 Mb per sample). All variants identified had at least a 50X coverage and more than 95% of these had more than 100x coverage. The sequencing results were analyzed by using the application SureCall 4.0 (Agilent, Santa Clara, CA, USA), adapted to the analysis of enriched Haloplex sequences, and MiseqReporter V04 (Illumina, San Diego, CA). Sequence revision against human genome was carried out by using the Integrative Genomes Viewer (Broad Institute, Cambridge, MA, USA).

#### Combined Dataset Analyses

All patients and controls participated in this phase. Analyses were carried out by using TaqMan genotyping focused on the SNVs raised after multiple comparison analyses of the NGS phase (see the results section for further details). The SNPs were analyzed in triplicate, by using SNP TaqMan assays (Life Technologies S.A., Alcobendas, Madrid, Spain), and following the conditions specified by the manufacturer. Assay details are as follows: FCER1G-rs36233990, Custom TaqMan® Assay; FCER1G-rs2070901, (C__15867981_20); and GC-rs3733359, (C__25652813_40).

### Statistical Analysis

The R package SNPasoc (Gonzalez et al., 2014) was used to calculate allele and genotypic frequencies, to determine the Hardy-Weinberg equilibrium using exact test (Wigginton, 2005) and to analyse differences between groups (González et al., 2007). The comparison between groups was performed with the Fisher's Exact Test (FET) and Likelihood Ratio Test (LRT) with an initial crude analysis followed by an adjusted analysis including gender as the categorical covariate when it was possible. False Discovery Rate (FDR) correction was used for the multiple comparison adjustments (Benjamini et al., 2001). The results were considered statistically significant when *P*-values were under 0.05. The association between SNPs and traits was estimated by odds ratio (OR) with a 95% confidence interval (CI) or by Relative Risk (RR) when the variation was not found in the control group. The Relative Risk was calculated by using EpiBasic, a tool for statistical analysis of tabular information, performing a stratified analysis, using the inverse variance (1/SE^2^) as weigh. This tool was developed as a companion to a Danish textbook on epidemiology (Juul, 2012), and the spreedsheat could be download from the following link (Juul and Frydenberg, 2016): http://ph.medarbejdere.au.dk/undervisning-og-uddannelse/software/.

Association between each SNP and each clinical phenotype was assessed by using binary logistic regression. Then, predictive Models based on Multinomial Logistic Regression (MLR) (Agresti, 2003) were performed for SNPs showing association in the binary regression analyses by using SPSS (IBM SPSS Statistics for Windows, Version 22.0). The *p*-values associated with every SNP were calculated using the Chi-Square test. Each model has associated several pseudo-R^2^ coefficients as indicators of the strength of the association between the response and the predictor variables. Cox and Snell is based on the loglikelihood for the model compared to the log likelihood for a baseline model and it has a theoretical maximum value of <1, even for a “perfect” model (Cox and Snell, 1989) and Nagelkerke is an adjusted version of the Cox & Snell *R*-square that adjusts the scale of the statistic to cover the full range from 0 to 1 (Nagelkerke, 1991). McFadden is another version, based on the log-likelihood kernels for the intercept-only model and the full estimated model. This is the pseudo-R^2^ coefficient most frequently used and the correlation between variables is good when the values are comprised between 0.2 and 0.4, and better up to 0.4 (McFadden, 1974, 1977). The first model includes all the groups separately, that is, AR+A, BLs, CR and SH. Model 2 considered two groups of patients: AR+A and a group combining all DRH patients. Model 3 considered three groups of patients: AR+A, patients with Ige-Mediated DHR, and patients with DHR not related to IgE (that is, CR patients). For all models the control group was always the reference group. Coefficients were calculated by dropping samples with missing data in explanatory variables, which have been selected using stepwise regression method. The statistical power was calculated from variant allele frequencies with a genetic model analysing the frequency for carriers of the disease gene with a RR value = 2 (*p* = 0.05) for the genetic associations identified in the combined dataset model as described elsewhere (Pértegas Díaz and Pita Fernández, 2003). These values are shown in Table S3. The functional impact of the gene variants was analyzed by using TRANSFAC (Matys et al., 2003, 2006).

## Results

### Identification of Novel Variants Using NGS

In this phase we identified 148 variations situated in the promoter region of genes related with vitamin D and FCεRI genes. The information about the variations, their frequency in the whole sample and the Hardy-Weinberg equilibrium values is summarized in Table 2. It is to be noted that 84 out of the 148 (56.7%) of the SNPs identified in this study were found in cases only and not in control individuals.

**Table 2:** SNPs with MAF ≥ 0.02 observed in the NGS study.

**Gene**	**dbSNP**	**Chromosomal Location**	**Alleles**	**MAF**	**HWE**	**MAF 1,000 Genomes**	**MAF 1,000 Genomes**	**MAF genomAD**	**MAF genomAD**
						**ALL individuals**	**European individuals**	**ALL individuals**	**European individuals**
FCER1A	rs2427837	1:159258545	G/A	0.213 A	0.501	0.151 A	0.304 A	0.217 A	0.289 A
FCER1A	rs61828219	1:159258641	C/A	0.141 A	0.752	0.085 A	0.160 A	0.129 A	0.175 A
FCER1A	rs12135235	1:159259029	T/G	0.043 G	1.000	0.033 G	0.033 G	0.034 G	0.037 G
FCER1G	rs36233990	1:161184658	C/T	0.020 T	0.134	0.011 T	0.006 T	0.010 T	0.013 T
FCER1G	None	1:161184792	GTCTCAAAAA/G	0.023 G	1.000	–	–	–	–
FCER1G	None	1:161184793	TCTCAAAAA/T	0.026 T	1.000	–	–	–	–
FCER1G	None	1:161184794	CTCAAAAAA/C	0.052 C	1.000	–	–	–	–
FCER1G	None	1:161184795	TCAAAAA/T	0.121 T	0.474	–	–	–	–
FCER1G	None	1:161184796	C/A	0.020 A	0.000	–	–	–	–
FCER1G	rs11587213	1:161184875	A/G	0.171 G	0.284	0.146 G	0.179 G	0.142 G	0.171 G
FCER1G	rs41270847	1:161184976	G/A	0.023 A	1.000	0.007 A	0.020 A	0.010 A	0.015 A
FCER1G	rs2070901	1:161185058	G/T	0.276 T	0.328	0.391 T	0.269 T	0.314 T	0.248 T
RXRG	rs3753897	1:165414511	C/A	0.152 A	0.082	0.215 A	0.195 A	0.211 A	0.193 A
RXRG	rs1467664	1:165414933	T/C	0.178 C	0.073	0.203 C	0.142 C	0.181 C	0.138 C
GC	rs3733359	4:72649774	G/A	0.069 A	0.894	0.206 A	0.055 A	0.122 A	0.060 A
GC	rs76781122	4:72669661	C/A	0.034 A	1.000	0.013 A	0.034 A	0.019 A	0.028 A
GC	rs6843222	4:72669944	C/T	0.029 T	1.000	0.004 T	0.016 T	0.007 T	0.011 T
GC	rs35096193	4:72670093	C/A	0.236 A	0.059	0.165 A	0.284 A	0.203 A	0.272 A
GC	rs1565572	4:72670191	A/C	0.210 C	0.499	0.432 C	0.196 C	0.363 C	0.192 C
GC	rs4020369	4:72670448	G/A	0.037 A	1.000	0.033 A	0	0.037 A	0.008 A
IL13	None	5:131992098	G/C	0.023 C	0.000	–	–	–	–
IL13	rs1800925	5:131992809	C/T	0.223 T	0.001	0.255 T	0.178 T	0.270 T	0.226 T
IL13	rs2066960	5:131994435	C/A	0.082 A	1.000	0.199 A	0.115 A	0.176 A	0.125 A
IL13	rs1295687	5:131994462	G/C	0.072 C	0.045	–	–	0.131 C	0.064 C
IL4	rs2243250	5:132009154	C/T	0.141 T	0.752	0.470 T	0.168 T	0.371 T	0.176 T
IL4	rs2070874	5:132009710	C/T	0.132 T	0.507	0.401 T	0.168 T	0.279 T	0.148 T
RXRB	rs76929655	6:33169182	T/C	0.020 C	1.000	0.002 C	0.005 C	0.007 C	0.011 C
CYP2R1	rs12794714	11:14913575	G/A	0.445 A	0.647	0.349 A	0.447 A	0.406 A	0.433 A
MS4A2	rs573790	11:59855385	C/T	0.382 T	1.000	0.441 T	0.356 T	0.460 T	0.411 T
MS4A2	rs574700	11:59855483	C/T	0.032 T	1.000	0.124 T	0.039 T	0.084 T	0.018 T
MS4A2	rs1441585	11:59855711	T/C	0.032 C	1.000	0.112 C	0.038 C	–	–
MS4A2	rs1441586	11:59856028	T/C	0.414 C	0.755	0.460 C	0.456 C	0.425 C	0.418 C
VDR	rs117397914	12:48276613	A/G	0.031 G	0.150	0.009 G	0.018 G	0.011 G	0.016 G
VDR	rs11168293	12:48293716	G/T	0.284 T	0.712	0.166 T	0.321 T	0.282 T	0.355 T
VDR	rs4303288	12:48336619	A/C	0.467 C	0.035	0.404 A	0.397 A	0.402 A	0.389 A
VDR	rs4307775	12:48336623	C/G	0.139 G	0.000	0.171 G	0.209 G	0.185 G	0.249 G
IL4R	rs12927172	16:27325021	G/A	0.424 A	0.013	0.405 A	0.372 A	0.353 A	0.367 A
IL4R	rs12927543	16:27325023	A/G	0.109 G	0.125	0.082 G	0.088 G	0.066 G	0.080 G
CYP24A1	rs35873579	20:52788190	G/A	0.023 A	1.000	0.001 A	0.004 A	0.002 A	0.003 A
CYP24A1	rs36106327	20:52788294	C/A	0.026 A	0.100	0.007 A	0.020 A	0.010 A	0.016 A
CYP24A1	rs2259735	20:52788314	T/C	0.455 C	0.029	0.566 C	0.420 C	0.515 C	0.408 C
CYP24A1	rs2762943	20:52790786	G/T	0.095 T	0.652	0.034 T	0.085 T	0.037 T	0.081 T
CYP24A1	rs2585427	20:52790976	G/C	0.376 C	0.873	0.444 C	0.390 C	0.447 C	0.373 C
IL13RA1	rs6603441	X:117861321	T/G	0.356 G	0.000	0.452 G	0.315 G	0.407 G	0.322 G

*-, not described; MAF, Minor Allele Frequency*.

Among the 148 gene variations identified, 43 were novel. Within the 105 already described SNPs, 25 have not been described or studied earlier in European individuals, although they show marginal MAF in our study (only three SNPs show MAF above 0.010). Regarding known SNPs, the frequencies are concordant with the results previously described in the 1,000 Genomes public database (http://grch37.ensembl.org/index.html) for individuals with European descent for all the variations except for the rs4020369 SNP in the *GC* gene, where the described frequency for Europeans is equal to 0, but in our population it shows a MAF close to 0.040, that is in agreement with the global frequency described in 1,000 Genomes for overall individuals.

One hundred and three out of the 148 variants identified were at Hardy-Weinberg equilibrium (HWE) in the overall study population (see Table S4), which is to be expected given the high number of SNPs analyzed and the limited sample size in the NGS analyses. Within the 45 variants that were not in HWE, only 7 showed a MAF>0.050 in agreement with frequencies described in literature. We carried out binary logistic regression analyses excluding those variants with MAF <0.02 (see Table 2). Results of the regression analyses are shown in Table S5. Among the 44 variants we selected those with adjusted *P* ≤ 0.10 for multinomial analyses. Therefore, 25 variants were included in the multinomial analysis as well as gender, antecedents of atopy and clinical group (Allergic rhinitis + Asthma; BLs, CR and SH). It is to be noted that some SNPs with a high significance after logistic binary regression analyses (See Table S5), such as rs1467664 (*RXRG*), rs3733359 (*GC*), rs2070874 (*IL4*), rs4303288, and rs4307775 (*VDR*) and rs2259735 (*CYP24A1)*, were not significant after multinomial analysis. The statistically significant variables raised after this analysis were three SNPs (*FCER1G* rs36233990, *FCER1G* rs2070901, and *GC* rs3733359), as well as antecedents of atopy.

### Combined Dataset Analyses

The three SNPs mentioned above were analyzed in the whole study group. The *FCER1G* rs36233990 SNP was monomorphic in the control group, whereas heterozygous subjects were identified in all subgroups of patients and homozygous individuals were identified in the AR+A and CR groups. Statistically significant differences were identified for this SNP in all subgroups of patients (Table 3), although after FDR correction differences remained significant for the AR+A and BLs groups. The *FCER1G* rs2070901 SNP had a marginal trend toward higher frequency of the variant allele among CR patients, which was not statistically significant after FDR analysis. These two FCER1G SNPs are not at linkage disequilibrium, being the D' value = 0.3415 and the *r* value = 0.0504. The minor allele frequency for the *GC* rs3733359 SNP was lower in the BLs group as compared to that of healthy individuals. This comparison remained statistically significant after FDR analysis.

**Table 3:** Combined dataset analyses.

			**Rhinits+Asthma (AR+A) vs. controls comparison values (*****N*** **=** **528 vs. 406)**			**Bls vs. Controls comparison values (*****N*** **=** **561 vs. 406)**			**CR vs. Controls comparison values (*****N*** **=** **668 vs. 406)**			**SH vs. Controls comparison values (*****N*** **=** **313 vs. 406)**		
**SNP ID**	**Genotype**	**Control frequency**	**AR+A frequency**	**OR (95% CI) with covariates (1) (2)**	**P_**cov**_ (FDR) (2)**	**BLs frequency**	**OR (95% CI) with covariates (1) (2)**	**P_**cov**_ (FDR) (2)**	**CR Frequency**	**OR (95% CI) with covariates (1) (2)**	**P_**cov**_ (FDR) (2)**	**SH Frequency**	**OR (95% CI) with covariates (1) (2)**	**P_**cov**_ (FDR) (2)**
FCER1G-rs36233990	C/C	1	0.953	1.00	0.000 (0.000)	0.991	1.00	0.025 (0.041)	0.990	1.00	0.043 (0.071)	0.993	1.00	0.077 (0.231)
	C/T	0	0.045	1.76 (1.66–1.86)		0.009	1.67 (1.53–1.76)		0.008	^*^		0.007	^*^	
	T/T	0	0.002	^*^		–	–		0.002	^*^		0	–	
FCER1G-rs2070901	G/G	0.482	0.531	1.00	0.065 (0.097)	0.545	1.00	0.073 (0.073)	0.524	1.00	0.047 (0.071)	0.521	1.00	0.328 (0.492)
	G/T	0.453	0.381	0.75 (0.56–0.99)		0.380	0.73 (0.55–0.96)		0.382	0.77 (0.59–1.01)		0.399	0.81 (0.59–1.11)	
	T/T	0.065	0.088	1.18 (0.69–2.00)		0.075	1.00 (0.58–1.72)		0.094	1.32 (0.80–2.20)		0.080	1.13 (0.62–2.06)	
GC-rs3733359	G/G	0.874	0.892	1.00	0.646 (0.646)	0.910	1.00	0.027 (0.041)	0.901	1.00	0.212 (0.212)	0.886	1.00	0.632 (0.632)
	G/A	0.118	0.104	0.88 (0.57–1.36)		0.090	0.73 (0.47–1.13)		0.098	0.79 (0.52–1.22)		0.110	0.92 (0.57–1.49)	
	A/A	0.008	0.004	0.50 (0.08–3.03)		0	0.00 (0.00–)		0.002	0.22 (0.02–2.10)		0.003	0.37 (0.04–3.63)	

Comparison between groups of patients and controls. (1) Relative Risk was calculated when the reference group (control) shows only non-mutated frequency. (2) OR and P-value were adjusted for gender. (

* *) OR adjusted by gender cannot be calculated due to lack of cases*.

The patients were grouped according to the underlying mechanism of the reaction. The first group was composed of all patients with drug hypersensitivity (DHR group), that is, (BLs + CR + SH). The second group of patients was composed of all patients with IgE-mediated drug hypersensitivity (D-IgE) (BLs + SH). The third group of patients was composed of all patients with IgE-mediated reactions (BLs + SH + AR+A), which were compared vs. healthy controls (Table 4). The two *FCER1G* SNPs displayed statistically significant differences in DHR and IgE-mediated reactions as compared to control individuals, although the only difference that remained significant after FDR correction was that of the SNP FCER1G rs2070901 in patients with IgE-mediated diseases (Table 4).

**Table 4:** Combined dataset analyses.

			**DHR vs. Control comparison values (*****N*** **=** **1,542 vs. 406)**			**DHR-IGE- vs. Control comparison values (*****N*** **=** **874 vs. 406)**			**All IGE-Mediated vs. Control comparison values (*****N*** **=** **1402 vs. 406)**		
**SNP ID**	**Genotype**	**Control Freq**.	**Drugs (BLs. CR. SH) Freq**	**OR (95% CI) with covariates (1) (2)**	**P_**cov**_ (FDR) (2)**	**Drugs IgE (BLs. SH) Freq**.	**OR (95% CI) with covariates (1) (2)**	**P_**cov**_ (FDR) (2)**	**IgE Mediated (AR+A. SH. BLs) Freq**.	**OR (95% CI) with covariates (1) (2)**	**P_**cov**_ (FDR) (2)**
FCER1G-rs36233990	C/C	1	0.991	1.00	0.047 (0.071)	0.992	1.00	0.025 (0.075)	0.977	1.00	0.001 (0.003)
	C/T	0	0.008	1.26 (1.23–1.29)		0.008	1.43 (1.38–1.49)		0.022	1.28 (1.25–1.31)	
	T/T	0	0.001	^*^		0	–		0.001	^*^	
FCER1G-rs2070901	G/G	0.482	0.531	1.00	0.044 (0.071)	0.536	1.00	0.089 (0.094)	0.534	1.00	0.047 (0.071)
	G/T	0.453	0.385	0.77 (0.61–0.97)		0.387	0.76 (0.59–0.98)		0.385	0.76 (0.60–0.96)	
	T/T	0.065	0.084	1.17 (0.74–1.87)		0.077	1.06 (0.64–1.74)		0.081	1.11 (0.69–1.77)	
GC-rs3733359	G/G	0.874	0.901	1.00	0.076 (0.076)	0.902	1.00	0.094 (0.094)	0.898	1.00	0.181 (0.181)
	G/A	0.118	0.097	0.80 (0.55–1.15)		0.097	0.80 (0.54–1.18)		0.100	0.83 (0.57–1.20)	
	A/A	0.008	0.001	0.17 (0.03–1.00)		0.001	0.14 (0.01–1.32)		0.002	0.26 (0.05–1.30)	

Comparisons depending on the mechanism. (1) Relative Risk was calculated when the reference group (control) shows only non-mutated frequency. (2) OR and P-value were adjusted for gender. (

* *) OR adjusted by gender cannot be calculated due to lack of cases*.

### Classification Models

We built models including the three SNPs with significant associations in the combined dataset analyses phase, as well as the antecedents of atopy and gender, and the pseudo R-square values for each model (those which provide the best classification of each patient in its correct group) are shown in Table 5. All the models selected the *FCER1G* rs36233990 as a good variable. The variable “Antecedents of atopy” was also selected, although this was expected because none of the control individuals had antecedents of atopy. Model 1 was made by including the three SNPs with a significant *P*-value and all the clinical groups separately, as compared to control subjects. Model 2 included all patients with DHR compared to control individuals, and model 3 included all patients with IgE-mediated diseases vs. control individuals. The classifications per group are shown in Table 6. It is to be noted the high percentage of correct allocations using the three SNPs only (that is, without considering antecedents of atopy and gender). For comparison, we show in Table 6 the results of the same models including covariables such as antecedents of atopy, age and gender. The fact that antecedents of atopy predicted 100% of AR+A patients has little value because control individuals had no antecedents. Age and gender, however, are not good predictors either (Table 6). Therefore, these covariables did not improve the predictive capacity of the models based on the SNPs.

**Table 5:** Statistically significant variables in each model and goodness-of-fit measures.

**Variables**	Model 1: Single groups model (*p*-value)^*^	Model 2: DHR Model (BLs, CR, SH)/Rhinitis+asthma/Control) (*p*-value)^*^	Model 3: IgE Mediated Drugs Model (BLs, SH)/Rhinitis+asthma/CR/Control) (*p*-value)^*^
*FCER1G* rs36233990	0.001	0.000	0.000
*FCER1G* rs2070901	0.520	0.169	0.337
*GC* rs3733359	0.810	0.517	0.766
Antecedents of atopy	0.030	0.000	0.000
Gender	0.000	0.106	0.137
**Pseudo R-square**			
Cox and Snell	0.522	0.520	0.521
Nagelkerke	0.546	0.606	0.557
McFadden	0.237	0.376	0.268

* *The overall effectiveness of the model was assessed by using the Chi-square statistic*.

**Table 6:** Prediction models.

**Observed (clinical classification)**	**Predicted (Group allocation according to the model) SNPs only (%) correct**	**Predicted (Group allocation according to the model) SNPs + Antecedents of atopy + gender+ age (%) correct**
AR+A (*n* = 481)	4.1	98.2
BLs (*n* = 230)	49.0	7.0
CR (*n* = 553)	53.4	68.6
SH (*n* = 300)	0	12.3
DHR (*n* = 1083)	99.0	81.8
DHR IgE-Mediated (*n* = 530)	99.2	49.6
All IgE-Mediated (*n* = 1,326)	99.9	79.6

## Discussion

Genetic variation is a major cause of interindividual differences in the susceptibility to a number of disorders. In this regard, a huge number of genetic association studies related to allergic disorders and drug hypersensitivity events have been carried out. Most of these studies have a case-control design interrogating only a few polymorphisms, typically, a few SNPs located within the coding region. The use of NGS techniques allows for a complete coverage of large areas thus revealing novel SNPs or analysing SNPs that are not included in most studies. In a previous NGS study in the promoter area of the genes encoding the COX-1 and COX-2 enzymes, we identified several novel SNPs. More than 70 SNPs modified transcription factor binding sites, either by disrupting existing sequences or by creating new binding sites (Agundez et al., 2014).

The present study is aimed to analyse the promoter areas of 16 genes related to allergic diseases and drug hypersensitivity reactions (DHRs). We have focused on the promoter gene region due to its crucial role in transcriptional activity and expression of the gene, as it has been observed for the SNPs located in the promoter of *FCER1A* (Potaczek et al., 2009), or *IL13* (Cameron et al., 2006; Kiesler et al., 2009; Li et al., 2014). The rationale for the selection of the 16 genes included in this study is based on putative mechanisms involved this type of reactions. FCERI plays an essential role in IgE-mediated mechanisms and variants in *FCERI* genes have been previously described as genetic factors related to asthma (Cui et al., 2003; Kim et al., 2006; Palikhe et al., 2008; Joubert et al., 2011; Ramphul et al., 2014; Yang et al., 2014), allergy (Hasegawa et al., 2003; de Guia et al., 2015; Liao et al., 2015; Amo et al., 2016a,b), and food sensitization (Liu et al., 2011; Hong and Wang, 2012). It has been also described that some variants in genes involved in the vitamin D pathway are related with asthma (Poon et al., 2004; Raby et al., 2004; Wjst, 2005; Wjst et al., 2006; Bossé et al., 2009; Saadi et al., 2009; Li et al., 2011; Pillai et al., 2011; Maalmi et al., 2013; Leung et al., 2015; Hutchinson et al., 2017), especially variations in genes regulated by vitamin D, such as *IL4* and its receptor (Burchard et al., 1999; Donfack et al., 2005; Ober and Hoffjan, 2006; Battle et al., 2007; Michel et al., 2010; Baye et al., 2011; Hesselmar et al., 2012; Liu et al., 2012; Micheal et al., 2013; Nie et al., 2013; Zhu et al., 2013; Al-Muhsen et al., 2014; Berenguer et al., 2014; Klaassen et al., 2015; Zhang et al., 2015; Narozna et al., 2016) and *IL13* (Black et al., 2009; Bottema et al., 2010; Palikhe et al., 2010; Cui et al., 2012; Accordini et al., 2016; Xu et al., 2017), which are also related to IgE (Marsh et al., 1994; Kabesch et al., 2006). According to previous research, the mechanisms involved in cross-reactions and selective ones are different, (Doña et al., 2012; Ayuso et al., 2013; Torres et al., 2014; Nissen et al., 2015; Amo et al., 2016a) and previously published results show that some variations, either related to *FCERI* or to vitamin D, are strongly associated with IgE-mediated pathologies, like rs12135235 in *FCER1A*, rs144205117 in *CYP2R1*, rs1467664 in *RXRG* or rs4303288 in *VDR*. Association between the rs2070874 in *IL4* and atopy and hypersensitivity, has been described in previously published works (Burchard et al., 1999; Donfack et al., 2005; Kabesch et al., 2006; Ober and Hoffjan, 2006; Kim et al., 2010; Madore and Laprise, 2010; Baye et al., 2011; Lu et al., 2011; Liu et al., 2012; Andiappan et al., 2013; Hsu et al., 2013; Micheal et al., 2013; Movahedi et al., 2013; Zhu et al., 2013; Berenguer et al., 2014; Caniatti et al., 2014; Li et al., 2014; de Guia et al., 2015; Klaassen et al., 2015; Zhang et al., 2015; Hua et al., 2016; Narozna et al., 2016).

Although binary logistic regression analyses pointed to six SNPs corresponding to *RXRG, GC, IL4, VDR*, and *CYP24A1* (see the Results section), statistical significance for these SNPs was not supported after multinomial analysis, except for the *GC* SNP. By turn, two additional *FCER1G* SNPs, as well as the *GC* SNP, were statistically significant after multinomial analyses. It is important to note that the major findings obtained in the present study are novel, since only one of the three SNPs that remained after the multinomial analysis have been previously related with atopy or drug hypersensitivity. Among these, one of the *FCER1G* SNPs is novel and hence have not been studied before, and the other one has been related with food sensitization (Liu et al., 2011) and has been previously studied in patients with selective hypersensitivity to NSAIDs and allergic rhinitis without significant association (Amo et al., 2016a,b). After the NGS and combined dataset analyses phases, prediction models revealed that one of these SNPs, designated as *FCER1G* rs36233990 was correct in all models and it allowed an excellent prediction for patients with DHR, IgE-mediated DHR and all IgE-mediated diseases analyzed. It should be taken into consideration that the significant *p*-values observed for rs36233990 in case-control association analyses might be inflated because this SNP was not observed in controls. However, this is a commonly observed SNP in European populations, which underscores the need for large control sets. This is a limitation in this study. The rs36233990 variation is located in a regulatory region where multiple transcription factor binding sites exist. The variant allele T triggers the appearance of E2F-3:Prrxl1 complex and GKLF (KLF4). On the other hand, the variant allele T leads the disappearance of a binding site for the transcription factor p300. Our own previous findings supported a role of *FCER* gene variations in patients with AR+A, but not in patients with SH (Park et al., 2011; Amo et al., 2016a) which are consistent with those raised in this study. The minor allele of rs2070901 in *FCERIG* triggers the disappearance of a transcription factor binding site for ELK-1: OC-2. The *GC* variation designated as rs3733359 is located in a splice region for transcripts 2 and 3 of GC, and in the 5' untranslated region for transcripts 1 and X1. This variant has been previously related to immune and other disorders (Jung et al., 2011; Wang et al., 2015; Xie et al., 2018). Our findings regarding the *GC* polymorphism support the hypothesis of a relevant role of vitamin D in allergy (Hall and Agrawal, 2017; Tian and Cheng, 2017.

In summary, our findings show that the analysis of the gene promoters is useful for the identification of genetic biomarkers of risk for DHRs and AR+A. Models using these gene variations allow a high degree of prediction, that is, correct group allocations (Table 6) based on these SNPs only. It should be kept in mind that the variant allele frequencies for these SNPs are relatively low, that is, the frequency of carriers of the risk variants is relatively low, specially for the most significant SNP *FCER1G* rs36233990 (<2% of patients). The frequencies of carriers for other two SNPs are 47 and 10.5% for *FCERIG* rs2070901 and *GC* rs3733359, respectively. Therefore, the presence of these gene variations cannot explain, by itself, the development of most cases of DHR. However, the SNPs raised in this study, point to mechanisms involved in DHR and add novel information that can be used as a proof of mechanism.

## Author Contributions

EG-M and JA contributed conception and design of the study. JG-M, CC, JC-G, NB-L, GC, ID, MB, and MT recruited and characterized patients. MM performed the statistical analysis. GA wrote the first draft of the manuscript. MM, EG-M, and JA wrote sections of the manuscript. All authors contributed to manuscript critical revision with important intellectual contribution, read, and approved the submitted version.

### Conflict of Interest Statement

The authors declare that the research was conducted in the absence of any commercial or financial relationships that could be construed as a potential conflict of interest.
