# Supplementary material for: Identification of Novel Biomarkers for Drug Hypersensitivity After Sequencing of the Promoter Area in 16 Genes of the Vitamin D Pathway and the High-Affinity IgE Receptor
Source: Front Genet. 2019 Jun 25;10:582. doi: 10.3389/fgene.2019.00582 (PMC6603231; doi:10.3389/fgene.2019.00582)
Supplement: Supplementary file 1 [file Table_1.pdf]

SUPPLEMENTAL MATERIAL

Table S1. Characteristics of the participants in the NGS phase.

	Healthy controls (n=22)	Patients with AR+A (n=22)	Patients with BLs hypersensitivity (n=43)	Patients with NSAIDs cross-reactions (CR) (n=41)	Patients with NSAIDs selective hypersensitivity (SH) (n=46)
Women. n (%)	14 (63.6%)	8 (36.4%)	22 (51.2%)	26 (63.4%)	29 (63.0%)
Age+sd (range)	23.9±8.8 (20-54)	28.9±12.3 (14-58)	43.5±14.8 (18-77)	43.2±15.7 (5-77)	45.3±14.2 (20-79)
Antecedents of atopy	0	22 (100%)	11 (25.6%)	14 (34.1%)	9 (19.6%)

Table S2. Details of the sequenced areas.

Gene-(protein coding transcript)	Coordinate: Origin	Coordinate: End
FCER1A-001	chr1:159258504	chr1:159259543
FCER1A-002	chr1:159272096	chr1:159272209
FCER1G-001, 002	chr1:161184111	chr1:161185160
RXRG-001	chr1:165414080	chr1:165415430
CYP27A1-001	chr2:219645905	chr2:219647160
GC-001,004, 007	chr4:72649678	chr4:72650079
GC-006	chr4:72669643	chr4:72670664
IL13-001	chr5:131991955	chr5:131995163
IL4-001, 002	chr5:132008742	chr5:132009877
RXRB-001, 002, 201, 202	chr6:33168019	chr6:33169254
RXRA-002	chr9:137217900	chr9:137218505
CYP2R1-001	chr11:14913527	chr11:14914751
MS4A2-001, 003	chr11:59855238	chr11:59856294
VDR-008	chr12:48276476	chr12:48276719
VDR-002,004,006, 007, 201, 202	chr12:48298340	chr12:48299414
VDR-001	chr12:48336470	chr12:48337000
CYP27B1-001	chr12:58160630	chr12:58161825
IL4R_001, 004, 006, 007, 009, 202, 203	chr16:27324989	chr16:27325562
CYP24A1-006	chr20:52788116	chr20:52788492
CYP24A1-003, 004	chr20:52789861	chr20:52791119
IL13RA1-001, 002	chrx:117860601	chrx:117861689

The coordinates for sequencing correspond to the GRCh37 assembly of the human genome.

Table S3. Statistical power for the genetic associations identified in this study.

	Patients with AR+A (Two-tailed)	Patients with BLs (Two-tailed)	Patients with NSAIDs cross-reactions (Two-tailed)	Patients with NSAIDs selective hypersensitivity (Two-tailed)	Patients with DHR (Two-tailed)	Patients with DHR-IGE (Two-tailed)	All IGE-mediated (Two-tailed)
FCER1G rs36233990	40.9%	43.0%	54.3%	32.3%	54.9%	47.6%	53.0 %
FCER1G rs2070901	>99%	>99%	>99%	>99%	>99%	>99%	>99%
GC rs3733359	97.5%	98.1%	98.8%	91.7%	>99%	>99%	>99%

TABLE S4. SNPs observed in the NGS study.

Gene	dbSNP	Chromosomal Location	Alleles	MAF	HWE	MAF 1000 Genomes ALL individuals	MAF 1000 Genomes European individuals	MAF genomAD ALL individuals	MAF genomAD European individuals
FCER1A	rs185716663	1:159258536	C/G	0.009 G	1.000	0.003 G	0.008 G	0.007 G	0.010 G
FCER1A	rs2427837	1:159258545	G/A	0.213 A	0.501	0.151 A	0.304 A	0.217 A	0.289 A
FCER1A	rs61828219	1:159258641	C/A	0.141 A	0.752	0.085 A	0.160 A	0.129 A	0.175 A
FCER1A	rs556527481	1:159258709	G/A	0.003 A	1.000	0.0002 A	0	0.0005 A	0.0005 A
FCER1A	rs190737507	1:159258798	A/G	0.003 G	1.000	0.001 G	0.003 G	0.002 G	0.003 G
FCER1A	rs12135235	1:159259029	T/G	0.043 G	1.000	0.033 G	0.033 G	0.034 G	0.037 G
FCER1A	None	1:159259290	A/C	0.003 C	1.000	-	-	-	-
FCER1A	rs746917868	1:159259461	A/C	0.003 C	1.000	-	-	0.001 C	0.0004 C
FCER1A	rs76362697	1:159259492	A/G	0.017 G	1.000	0.009 G	0.026 G	0.014 G	0.020 G
FCER1A	rs983088525	1:159272158	G/T	0.003 T	1.000	-	-	0.000004 T	0
FCER1A	rs200728340	1:159272209	G/A	0.003 A	1.000	-	-	0.00004 A	0.00005 A
FCER1G	rs77823804	1:161184213	T/G	0.006 G	1.000	0.002 G	0.006 G	0.002 G	0.003 G
FCER1G	rs567742742	1:161184367	G/A	0.003 A	1.000	-	-	0.0006 A	0.001 A
FCER1G	rs36233990	1:161184658	C/T	0.020 T	0.134	0.011 T	0.006 T	0.010 T	0.013 T
FCER1G	rs750727758	1:161184669	G/T	0.006 T	0.003	-	-	-	-
FCER1G	rs150957027	1:161184763	C/T	0.003 T	1.000	0.002 T	0	0.001 T	0.0001 T
FCER1G	None	1:161184792	GTCTCAAAAA/G	0.023 G	1.000	-	-	-	-
FCER1G	None	1:161184793	TCTCAAAAA/T	0.026 T	1.000	-	-	-	-
FCER1G	None	1:161184794	CTCAAAAAA/C	0.052 C	1.000	-	-	-	-
FCER1G	None	1:161184795	TCAAAAA/T	0.121 T	0.474	-	-	-	-
FCER1G	None	1:161184796	C/A	0.020 A	0.000	-	-	-	-
FCER1G	rs11587213	1:161184875	A/G	0.171 G	0.284	0.146 G	0.179 G	0.142 G	0.171 G
FCER1G	None	1:161184964	C/T	0.003 T	1.000	-	-	-	-
FCER1G	rs41270847	1:161184976	G/A	0.023 A	1.000	0.007 A	0.020 A	0.010 A	0.015 A
FCER1G	rs183879788	1:161185037	G/A	0.006 A	1.000	0.001 A	0.002 A	0.0007 A	0.001 A
FCER1G	rs2070901	1:161185058	G/T	0.276 T	0.328	0.391 T	0.269 T	0.314 T	0.248 T
RXRG	rs61800591	1:165414180	C/T	0.011 T	1.000	0.004 T	0.018 T	0.014 T	0.021 T
RXRG	rs57637440	1:165414267	C/A	0.003 A	1.000	0.008 A	0	0.008 A	0.00006 A
RXRG	None	1:165414386	ATGC/A	0.006 A	1.000	-	-	-	-
RXRG	rs3753897	1:165414511	C/A	0.152 A	0.082	0.215 A	0.195 A	0.211 A	0.193 A
RXRG	rs114530659	1:165414855	A/C	0.003 C	1.000	0.037 C	0.003 C	0.025 C	0.005 C
RXRG	rs1467664	1:165414933	T/C	0.178 C	0.073	0.203 C	0.142 C	0.181 C	0.138 C
RXRG	None	1:165415140	G/C	0.003 C	1.000	-	-	-	-
RXRG	None	1:165415222	A/G	0.003 G	1.000	-	-	-	-
CYP27A1	rs147477169	2:219646129	T/C	0.009 C	1.000	0.003 C	0.011 C	0.011 C	0.015 C
CYP27A1	rs114345604	2:219646470	T/C	0.006 C	1.000	0.014 C	0	0.013 C	0.0002 C
CYP27A1	rs182545847	2:219646536	T/C	0.006 C	1.000	0.0002 C	0	0.005 C	0.007 C
CYP27A1	None	2:219646547	AC/A	0.017 A	0.000	-	-	-	-
CYP27A1	rs778193906	2:219646962	C/T	0.006 T	0.003	-	-	0.000007 A	0
CYP27A1	rs757653354	2:219646967	G/T	0.006 T	0.003	-	-	0.000007 A	0
GC	rs3733359	4:72649774	G/A	0.069 A	0.894	0.206 A	0.055 A	0.122 A	0.060 A

Gene	dbSNP	Chromosomal Location	Alleles	MAF	HWE	MAF 1000 Genomes ALL individuals	MAF 1000 Genomes European individuals	MAF genomAD ALL individuals	MAF genomAD European individuals
GC	rs76781122	4:72669661	C/A	0.034 A	1.000	0.013 A	0.034 A	0.019 A	0.028 A
GC	rs6843222	4:72669944	C/T	0.029 T	1.000	0.004 T	0.016 T	0.007 T	0.011 T
GC	rs1565571	4:72670025	C/T	0.011 T	0.000	0.002 T	0	0.002 T	0
GC	rs35096193	4:72670093	C/A	0.236 A	0.059	0.165 A	0.284 A	0.203 A	0.272 A
GC	rs1565572	4:72670191	A/C	0.210 C	0.499	0.432 C	0.196 C	0.363 C	0.192 C
GC	rs2365085	4:72670432	T/A	0.003 A	1.000	0.033 A	0.001 A	0.038 A	0.009 A
GC	rs113387725	4:72670436	G/A	0.003 A	1.000	0.155 A	0.059 A	0.099 A	0.057 A
GC	rs4020369	4:72670448	G/A	0.037 A	1.000	0.033 A	0	0.037 A	0.008 A
IL13	rs186788419	5:131991999	G/C	0.014 C	0.000	-	-	0.0005 C	0.0007 C
IL13	rs544266451	5:131992049	T/C	0.011 C	0.000	-	-	-	-
IL13	None	5:131992053	C/T	0.006 T	0.003	-	-	-	-
IL13	None	5:131992056	G/A	0.011 A	0.000	-	-	-	-
IL13	None	5:131992060	A/C	0.011 C	0.003	-	-	-	-
IL13	None	5:131992064	C/T	0.006 T	0.003	-	-	-	-
IL13	None	5:131992065	T/C	0.006 C	0.003	-	-	-	-
IL13	None	5:131992067	C/A	0.006 A	0.003	-	-	-	-
IL13	rs1010937360	5:131992068	C/T	0.006 T	0.003	-	-	-	-
IL13	None	5:131992073	G/C	0.006 C	0.003	-	-	-	-
IL13	None	5:131992080	C/T	0.006 T	0.003	-	-	-	-
IL13	None	5:131992098	G/C	0.023 C	0.000	-	-	-	-
IL13	None	5:131992103	G/C	0.006 C	0.003	-	-	-	-
IL13	None	5:131992115	G/A	0.011 A	0.000	-	-	-	-
IL13	None	5:131992117	A/C	0.006 C	0.003	-	-	-	-
IL13	None	5:131992118	G/A	0.006 A	0.003	-	-	-	-
IL13	None	5:131992120	T/C	0.014 C	0.000	-	-	-	-
IL13	None	5:131992126	T/C	0.011 C	0.000	-	-	-	-
IL13	None	5:131992127	T/C	0.006 C	0.003	-	-	-	-
IL13	None	5:131992129	T/C	0.006 C	0.003	-	-	-	-
IL13	None	5:131992197	C/T	0.006 T	0.003	-	-	-	-
IL13	None	5:131992229	C/T	0.011 T	0.000	-	-	-	-
IL13	None	5:131992241	T/C	0.011 C	0.000	-	-	-	-
IL13	None	5:131992243	C/A	0.011 A	0.000	-	-	-	-
IL13	rs770116349	5:131992295	C/T	0.003 T	1.000	-	-	0.00006 T	0.0001 T
IL13	None	5:131992807	A/C	0.006 C	0.003	-	-	-	-
IL13	rs1800925	5:131992809	C/T	0.223 T	0.001	0.255 T	0.178 T	0.270 T	0.226 T
IL13	rs2066960	5:131994435	C/A	0.082 A	1.000	0.199 A	0.115 A	0.176 A	0.125 A
IL13	rs528784778	5:131994444	G/A	0.003 A	1.000	0.0002 A	0.001 A	-	-
IL13	rs1295687	5:131994462	G/C	0.072 C	0.045	-	-	0.131 C	0.064 C
IL13	rs2069744	5:131994669	C/T	0.014 T	1.000	0.124 T	0.010 T	0.091 T	0.004 T
IL13	rs574210453	5:131994954	A/G	0.009 G	1.000	0.0004 G	0.001 G	0.0002 G	0
IL13	rs533616846	5:131994965	G/T	0.006 T	1.000	0.0004 T	0.001 T	0.0001 T	0.0001 T
IL13	rs2069746	5:131995079	C/T	0.003 T	1.000	0.013 T	0	0.003 T	0.0001 T
IL4	rs145217073	5:132008886	G/C	0.006 C	1.000	0	0.001 C	0.002 C	0.002 C
IL4	rs752517771	5:132009033	G/C	0.006 C	0.003	-	-	-	-
IL4	rs2243250	5:132009154	C/T	0.141 T	0.752	0.470 T	0.168 T	0.371 T	0.176 T
IL4	rs894692486	5:132009374	T/C	0.003 C	1.000	-	-	0.00003 C	0.00006 C
IL4	rs539499377	5:132009579	T/A	0.003 A	1.000	0.0002 A	0	-	-
IL4	rs17772853	5:132009599	C/T	0.006 T	0.003	0.010 T	0.011 T	0.008 T	0.004 T
IL4	rs2070874	5:132009710	C/T	0.132 T	0.507	0.401 T	0.168 T	0.279 T	0.148 T
IL4	rs2243251	5:132009787	A/G	0.009 G	1.000	0.059 G	0.002 G	0.017 G	0.0007 G

Gene	dbSNP	Chromosomal Location	Alleles	MAF	HWE	MAF 1000 Genomes ALL individuals	MAF 1000 Genomes European individuals	MAF genomAD ALL individuals	MAF genomAD European individuals
RXRB	rs376711309	6:33168304	G/T	0.003 T	1.000	0.0002 T	0.001 T	0.0005 T	0.0008 T
RXRB	rs577415071	6:33168901	T/C	0.003 C	1.000	0.0002 C	0.001 C	0.0004 C	0.0008 C
RXRB	rs76929655	6:33169182	T/C	0.020 C	1.000	0.002 C	0.005 C	0.007 C	0.011 C
RXRB	rs772036010	6:33169223	T/G	0.006 G	0.003	-	-	0.000004 C	0
CYP2R1	rs12794714	11:14913575	G/A	0.445 A	0.647	0.349 A	0.447 A	0.406 A	0.433 A
CYP2R1	rs187639972	11:14913900	G/C	0.003 C	1.000	0.001 C	0.003 C	0.001 C	0.002 C
CYP2R1	None	11:14914082	A/C	0.006 C	0.003	-	-	-	-
CYP2R1	rs144205117	11:14914601	C/A	0.012 A	1.000	0.030 A	0.010 A	0.008 A	0.009 A
MS4A2	rs573790	11:59855385	C/T	0.382 T	1.000	0.441 T	0.356 T	0.460 T	0.411 T
MS4A2	rs574700	11:59855483	C/T	0.032 T	1.000	0.124 T	0.039 T	0.084 T	0.018 T
MS4A2	rs756668901	11:59855532	T/G	0.003 G	1.000	-	-	0.0002 G	0.0004 G
MS4A2	rs780370785	11:59855542	T/C	0.003 C	1.000	-	-	0.0001 C	0.0001 C
MS4A2	rs1441585	11:59855711	T/C	0.032 C	1.000	0.112 C	0.038 C	-	-
MS4A2	None	11:59855918	T/C	0.003 C	1.000	-	-	-	-
MS4A2	rs73479087	11:59855960	A/G	0.003 G	1.000	0.015 G	0	0.011 G	0.0004 G
MS4A2	rs1441586	11:59856028	T/C	0.414 C	0.755	0.460 C	0.456 C	0.425 C	0.418 C
MS4A2	rs920168033	11:59856086	T/C	0.003 C	1.000	-	-	-	-
MS4A2	rs140124027	11:59856240	T/G	0.003 G	1.000	-	-	0.0002 G	0.0003 G
VDR	rs117397914	12:48276613	A/G	0.031 G	0.150	0.009 G	0.018 G	0.011 G	0.016 G
VDR	rs11168293	12:48293716	G/T	0.284 T	0.712	0.166 T	0.321 T	0.282 T	0.355 T
VDR	None	12:48293730	G/A	0.003 A	1.000	-	-	-	-
VDR	None	12:48298652	C/A	0.003 A	1.000	-	-	-	-
VDR	rs144185611	12:48299279	G/T	0.003 T	1.000	0.001 T	0.005 T	0.003 T	0.002 T
VDR	None	12:48336499	T/A	0.003 A	1.000	-	-	-	-
VDR	rs4303288	12:48336619	A/C	0.467 C	0.035	0.404 A	0.397 A	0.402 A	0.389 A
VDR	rs4307775	12:48336623	C/G	0.139 G	0.000	0.171 G	0.209 G	0.185 G	0.249 G
VDR	rs138142537	12:48336736	A/C	0.006 C	1.000	0.007 C	0.015 C	0.008 C	0.012 C
CYP27B1	None	12:58160838	G/C	0.003 C	1.000	-	-	-	-
CYP27B1	rs529566631	12:58161026	A/G	0.003 G	1.000	-	-	0.001 G	0.001 G
CYP27B1	rs117864436	12:58161242	C/T	0.011 T	1.000	0.002 T	0.006 T	0.005 T	0.009 T
CYP27B1	rs148627073	12:58161599	G/A	0.014 A	0.029	0.004 A	0.015 A	0.003 A	0.005 A
CYP27B1	None	12:58161746	G/A	0.006 A	0.003	-	-	-	-
IL4R	rs12927172	16:27325021	G/A	0.424 A	0.013	0.405 A	0.372 A	0.353 A	0.367 A
IL4R	rs12927543	16:27325023	A/G	0.109 G	0.125	0.082 G	0.088 G	0.066 G	0.080 G
IL4R	rs55919742	16:27325028	G/A	0.006 A	1.000	0.013 A	0.002 A	0.011 A	0.001 A
CYP24A1	rs35873579	20:52788190	G/A	0.023 A	1.000	0.001 A	0.004 A	0.002 A	0.003 A
CYP24A1	rs36106327	20:52788294	C/A	0.026 A	0.100	0.007 A	0.020 A	0.010 A	0.016 A
CYP24A1	rs2259735	20:52788314	T/C	0.455 C	0.029	0.566 C	0.420 C	0.515 C	0.408 C
CYP24A1	rs17219315	20:52788446	A/G	0.014 G	1.000	0.009 G	0.018 G	0.013 G	0.019 G
CYP24A1	rs59262760	20:52789417	C/T	0.006 T	1.000	0.066 T	0	0.022 T	0.002 T
CYP24A1	None	20:52789466	CCTT/C	0.006 C	1.000	-	-	-	-
CYP24A1	rs61755338	20:52789885	A/C	0.014 C	1.000	0.009 C	0.018 C	0.016 C	0.022 C
CYP24A1	rs61749689	20:52790005	C/A	0.017 A	1.000	0.008 A	0.018 A	0.016 A	0.023 A
CYP24A1	rs73913755	20:52790194	G/A	0.006 A	1.000	0.050 A	0	0.046 A	0.001 A
CYP24A1	rs144737632	20:52790218	A/G	0.017 G	1.000	0.008 G	0.018 G	0.012 G	0.019 G
CYP24A1	None	20:52790469	GTC/G	0.006 G	1.000	-	-	-	-
CYP24A1	None	20:52790470	TC/T	0.011 T	1.000	-	-	-	-
CYP24A1	rs73913757	20:52790518	C/T	0.006 T	1.000	0.049 T	0	0.044 T	0.001 T
CYP24A1	rs536169079	20:52790704	G/C	0.014 C	1.000	0.008 C	0.018 C	0.012 C	0.019 C
CYP24A1	rs373075992	20:52790754	C/T	0.014 T	1.000	0.008 T	0.018 T	0.012 T	0.019 T

Gene	dbSNP	Chromosomal Location	Alleles	MAF	HWE	MAF 1000 Genomes ALL individuals	MAF 1000 Genomes European individuals	MAF genomAD ALL individuals	MAF genomAD European individuals
CYP24A1	rs2762943	20:52790786	G/T	0.095 T	0.652	0.034 T	0.085 T	0.037 T	0.081 T
CYP24A1	rs2585427	20:52790976	G/C	0.376 C	0.873	0.444 C	0.390 C	0.447 C	0.373 C
CYP24A1	rs187147516	20:52790984	C/A	0.014 A	1.000	0.010 A	0.024 A	0.015 A	0.023 A
IL13RA1	rs141993027	X:117860913	T/C	0.003 C	1.000	0.001 C	0	0.001 C	0
IL13RA1	rs187367085	X:117861106	G/A	0.003 A	1.000	0.001 A	0.003 A	0.0006 A	0.0004 A
IL13RA1	rs6603441	X:117861321	T/G	0.356 G	0.000	0.452 G	0.315 G	0.407 G	0.322 G
IL13RA1	rs555458472	X:117861629	C/T	0.006 T	0.003	-	-	0.00004 T	0

-: not described; MAF: Minor Allele Frequency.

Table S5. Adjusted *P*-values after logistic binary regression analyses of SNPs with MAF ≥ 0.02 observed in the NGS study.

SNP	Chromosomal location	R+A vs controls	BLs vs Controls	CR vs Controls	SH vs Controls	Drugs-Controls	Drugs IgE-Controls	IgE med-Controls	R+A vs rest of patients	BLs vs rest of patients	CR vs rest of patients	SH vs rest of patients
rs2427837	1:159258545	0.240	0.121	0.464	0.278	0.289	0.228	0.213	0.568	0.200	0.311	0.857
rs61828219	1:159258641	0.238	0.780	0.294	0.865	0.749	0.885	0.764	0.437	0.787	0.413	0.370
rs12135235	1:159259029	0.045	0.043	0.101	0.071	0.049	0.042	0.034	0.677	0.685	0.552	0.800
rs36233990	1:161184658	0.144	0.075	0.037	0.070	0.014	0.024	0.015	0.514	0.894	0.182	0.822
None	1:161184792	0.335	0.951	0.293	0.130	0.857	0.545	0.431	0.123	0.959	0.012	0.021
None	1:161184793	0.335	0.051	0.185	0.374	0.117	0.112	0.134	0.538	0.096	0.883	0.215
None	1:161184794	0.147	0.080	0.003	0.072	0.018	0.060	0.071	0.192	0.512	0.022	0.444
None	1:161184795	0.238	0.338	0.198	0.009	0.037	0.041	0.035	0.809	0.039	0.336	0.009
None	1:161184796	0.383	0.153	-	-	0.411	0.320	0.485	0.141	0.251	0.285	0.236
rs11587213	1:161184875	0.169	0.340	0.880	0.514	0.557	0.328	0.224	0.355	0.745	0.183	0.982
rs41270847	1:161184976	1.000	0.817	0.447	0.124	0.988	0.488	0.547	0.959	0.983	0.085	0.022
rs2070901	1:161185058	0.626	0.226	0.315	0.056	0.103	0.074	0.097	0.801	0.593	0.666	0.284
rs3753897	1:165414511	0.792	0.312	0.645	0.188	0.296	0.209	0.273	0.317	0.650	0.551	0.357
rs1467664	1:165414933	0.007	0.178	0.256	0.026	0.105	0.098	0.053	0.023	0.017	0.484	0.365
rs3733359	4:72649774	0.571	0.318	0.342	0.092	0.224	0.170	0.174	0.725	0.529	0.106	0.008
rs76781122	4:72669661	1.000	0.922	0.523	0.435	0.623	0.693	0.744	0.626	0.399	0.579	0.432
rs6843222	4:72669944	0.425	0.507	0.226	0.690	0.436	0.588	0.520	0.613	0.636	0.382	0.524
rs35096193	4:72670093	0.626	0.393	0.405	0.820	0.514	0.586	0.637	0.341	0.626	0.412	0.530
rs1565572	4:72670191	0.724	0.815	0.508	0.708	0.683	0.794	0.761	0.988	0.785	0.684	0.519
rs4020369	4:72670448	-	0.197	0.018	0.042	0.035	0.068	0.094	0.062	0.290	0.157	0.608
None	5:131992098	0.335	0.197	-	0.371	0.327	0.241	0.229	0.626	0.360	0.113	0.830
rs1800925	5:131992809	0.210	0.335	0.712	0.489	0.454	0.363	0.334	0.238	0.744	0.667	0.431
rs2066960	5:131994435	0.336	0.903	0.612	0.270	0.699	0.492	0.753	0.144	0.724	0.302	0.207
rs1295687	5:131994462	0.095	0.444	0.392	0.554	0.559	0.432	0.436	0.085	0.342	0.944	0.249
rs2243250	5:132009154	0.036	0.114	0.022	0.329	0.110	0.276	0.181	0.266	0.250	0.209	0.123
rs2070874	5:132009710	0.019	0.024	0.008	0.159	0.032	0.091	0.060	0.443	0.277	0.271	0.185
rs76929655	6:33169182	0.335	0.197	0.179	0.203	0.170	0.170	0.169	0.857	0.979	0.870	0.974
rs12794714	11:14913575	0.685	0.424	0.729	0.742	0.767	0.715	0.775	0.610	0.153	0.727	0.910
rs573790	11:59855385	0.071	0.089	0.038	0.225	0.094	0.146	0.107	0.265	0.743	0.134	0.830
rs574700	11:59855483	0.081	0.552	0.802	0.949	0.699	0.690	0.512	0.102	0.639	0.614	0.316
rs1441585	11:59855711	0.081	0.552	0.802	0.949	0.699	0.690	0.512	0.102	0.639	0.614	0.316
rs1441586	11:59856028	0.260	0.487	0.279	0.881	0.544	0.678	0.594	0.496	0.891	0.250	0.507
rs117397914	12:48276613	0.147	0.946	0.447	0.670	0.807	0.782	0.781	0.319	0.724	0.268	0.318
rs11168293	12:48293716	0.535	0.573	0.146	0.047	0.155	0.208	0.184	0.891	0.131	0.807	0.109

rs4303288	12:48336619	0.288	0.011	0.726	0.884	0.298	0.193	0.211	0.284	0.002	0.414	0.016
rs4307775	12:48336623	0.014	0.001	0.002	0.000	0.000	0.000	0.000	0.994	0.635	0.639	0.925
rs12927172	16:27325021	0.490	0.831	0.906	0.171	0.572	0.403	0.716	0.113	0.970	0.616	0.077
rs12927543	16:27325023	0.394	0.931	0.895	0.869	0.862	0.890	0.811	0.181	0.957	0.960	0.761
rs35873579	20:52788190	0.425	0.743	0.953	0.976	0.908	0.864	0.919	0.145	0.957	0.960	0.761
rs36106327	20:52788294	0.097	0.237	0.095	0.130	0.079	0.166	0.096	0.041	0.394	0.394	0.019
rs2259735	20:52788314	0.003	0.000	0.000	0.000	0.000	0.000	0.000	0.567	0.625	0.630	0.308
rs2762943	20:52790786	0.453	0.547	0.716	0.647	0.647	0.556	0.633	0.492	0.05	0.471	0.897
rs2585427	20:52790976	0.287	0.856	0.595	0.562	0.622	0.651	0.526	0.420	0.702	0.762	0.975
rs6603441	X:117861321	0.251	0.295	0.054	0.318	0.124	0.272	0.190	0.451	0.354	0.311	0.781
